# Suppression of Conditional TDP-43 Transgene Expression Differentially Affects Early Cognitive and Social Phenotypes in TDP-43 Mice

**DOI:** 10.3389/fgene.2019.00369

**Published:** 2019-04-24

**Authors:** Pablo R. Silva, Gabriela V. Nieva, Lionel M. Igaz

**Affiliations:** IFIBIO Bernardo Houssay, Grupo de Neurociencia de Sistemas, Facultad de Medicina, Universidad de Buenos Aires-CONICET, Buenos Aires, Argentina

**Keywords:** TDP-43, frontotemporal dementia, amyotrophic lateral sclerosis, transgenic mice, behavior, animal model, proteinopathy

## Abstract

Dysregulation of TAR DNA-binding protein 43 (TDP-43) is a hallmark feature of frontotemporal dementia (FTD) and amyotrophic lateral sclerosis (ALS), two fatal neurodegenerative diseases. TDP-43 is a ubiquitously expressed RNA-binding protein with many physiological functions, playing a role in multiple aspects of RNA metabolism. We developed transgenic mice conditionally overexpressing human wild-type TDP-43 protein (hTDP-43-WT) in forebrain neurons, a model that recapitulates several key features of FTD. After post-weaning transgene (TG) induction during 1 month, these mice display an early behavioral phenotype, including impaired cognitive and social function with no substantial motor abnormalities. In order to expand the analysis of this model, we took advantage of the temporal and regional control of TG expression possible in these mice. We behaviorally evaluated mice at two different times: after 2 weeks of post-weaning TG induction (0.5 month group) and after subsequent TG suppression for 2 weeks following that time point [1 month (sup) group]. We found no cognitive abnormalities after 0.5 month of hTDP-43 expression, evaluated with a spatial working memory task (Y-maze test). Suppression of TG expression with doxycycline (Dox) at this time point prevented the development of cognitive deficits previously observed at 1 month post-induction, as revealed by the performance of the 1 month (sup) group. On the other hand, sociability deficits (assessed through the social interaction test) appeared very rapidly after Dox removal (0.5 month) and TG suppression was not sufficient to reverse this phenotype, indicating differential vulnerability to hTDP-43 expression and suppression. Animals evaluated at the early time point (0.5 month) post-induction do not display a motor phenotype, in agreement with the results obtained after 1 month of TG expression. Moreover, all motor tests (open field, accelerated rotarod, limb clasping, hanging wire grip) showed identical responses in both control and bigenic animals in the suppressed group, demonstrating that this protocol and treatment do not cause non-specific effects in motor behavior, which could potentially mask the phenotypes in other domains. Our results show that TDP-43-WT mice have a phenotype that qualifies them as a useful model of FTD and provide valuable information for susceptibility windows in therapeutic strategies for TDP-43 proteinopathies.

## Introduction

Neurodegenerative diseases are incurable and debilitating conditions that arise as a consequence of the progressive degeneration and/or death of nerve cells. This heterogeneous group of disorders is characterized by behavioral changes that differ according to the disease entity. Among these, most forms of amyotrophic lateral sclerosis (ALS) and frontotemporal dementia (FTD) show clinic-pathological overlap. These diseases may represent a single pathological entity with diverse clinical manifestations (Geser et al., 2010; Burrell et al., 2016), included within the heterogeneous group of “TDP-43 proteinopathies.” TDP-43 pathology frequently associates with other disorders, including Alzheimer’s disease, dementia with Lewy body, hippocampal sclerosis, and chronic traumatic encephalopathy, among others (Kovacs, 2016). The umbrella term “TDP-43 proteinopathies” was coined shortly after the discovery that most forms of ALS and around 45% of FTD cases have TDP-43-positive neuronal and glial inclusions as a major pathological hallmark (Arai et al., 2006; Neumann et al., 2006; Cairns et al., 2007). Roughly, another 45% of FTD cases is characterized with tau pathology and 5–10% with FUS accumulation, while a small percentage of ALS cases is associated with abnormalities in SOD1, FUS, or other proteins (Nguyen et al., 2018).

TDP-43 is a highly conserved and widely expressed RNA-binding protein (RBP) that normally resides predominantly in the nucleus of all cells. It has been described to be involved in different cellular processes, most conspicuously RNA metabolism, including RNA translation, splicing, and transport (Ratti and Buratti, 2016). Dysregulation of RNA metabolism can occur at multiple levels of RNA processing including transcription, splicing, mRNA transport, stability, and translation (Coyne et al., 2017). This, in turn, will have numerous implications for the generation of biochemical, pathological, and behavioral phenotypes. Although several animal models of FTD/ALS disease have been developed in the past few years, an important caveat is that none exactly mimic the pathophysiology and the phenotype of human FTD/ALS (Alrafiah, 2018). However, studying the behavioral impact of modulating FTD/ALS-related RBPs shows that they recapitulate different clinical presentations in patients, representing an array of behavioral domains that include motor, cognitive, and social symptoms (Philips and Rothstein, 2015; Nolan et al., 2016; Ahmed et al., 2017; Tan et al., 2017; Ittner et al., 2018).

Given that multiple (although not all) forms of both sporadic and familial FTD/ALS cases of different genetic origin (i.e., mutations in C9orf72, progranulin, TARDBP, etc.) converge in a common pathological presentation that involves TDP-43 dysregulation (Seelaar et al., 2011; Renton et al., 2014), there is a growing need in the field for understanding the pathological and behavioral consequences of these abnormalities.

Using a mouse model of TDP-43 proteinopathies that conditionally overexpresses the wild-type human TDP-43 protein (hTDP-43-WT) in forebrain neurons and reproduces neuropathological changes of the FTD/ALS spectrum (Igaz et al., 2011), we have recently shown that they display early behavioral phenotypes in the cognitive and social domains (Alfieri et al., 2016). Interestingly, these animals also exhibit progressive motor abnormalities after prolonged transgene expression (Alfieri et al., 2016). These behavioral features provide an interesting correlate to human disease, starting with a more “pure” FTD-like phenotype and later evolving into a FTD with motor neuron disease presentation (expressing some ALS-like features). A major advantage of using inducible transgenic systems is the possibility to prevent or reverse specific phenotypes after transgene suppression. This, in turn, may provide information on the differential susceptibility of the diverse phenotypical manifestations of TDP-43 manipulation, with implications for understanding pathological onset and progression in human disease and defining time windows with therapeutic relevance.

In this work, we aimed to investigate how short-term transgene suppression affects the early behavioral phenotypes displayed by conditional TDP-43-WT mice (Alfieri et al., 2016). In particular, we studied (1) if the cognitive and social phenotypes previously described were present after a shorter period (0.5 month) of transgene expression, and (2) the effect of the suppression on both affected domains and in motor behaviors, which were preserved after 1 month of transgene expression. Assessment of the different behavioral domains both before and after transgene suppression indicates that sociability is rapidly impaired while cognitive and motor performance is preserved after 0.5 month of hTDP-43 expression. Moreover, suppression of transgene expression prevented the development of cognitive deficits and had no effect on motor behavior. Remarkably, social behavior remained compromised after transgene suppression, indicating differential vulnerability to TDP-43 manipulation in the neuronal circuits underlying diverse behaviors.

These results emphasize the need to comprehensively evaluate the behavioral phenotypes in multiple disease models and suggest that the timing of treatment could widely influence the outcome of the different clinical manifestations of TDP-43 proteinopathies.

## Materials and Methods

### Animals

This study was carried out in accordance with the recommendations of the National Animal Care and Use Committee of the University of Buenos Aires (CICUAL). The protocol was approved by the CICUAL. Mice were kept under a 12-h light/dark cycle, with controlled temperature (23 ± 2°C) and humidity (40–60%) and had *ad libitum* access to food and water. To produce hTDP-43 transgenic lines, as described previously (Igaz et al., 2011), pronucleus of fertilized eggs from C57BL/6J × C3HeJ F1 matings were injected with a vector containing hTDP-43-WT cDNA. Monogenic tetO-TDP-WT12 mice were bred to Camk2a-tTA mice (Mayford et al., 1996; Jackson Laboratory) generating non-transgenic, tTA monogenic, single tetO-TDP-43 transgenic mice (non-TDP-43 expressing control mice) and bigenic mice expressing hTDP-43-WT12 (hereinafter referred to as tTA/WT12).

**Figure 1:**
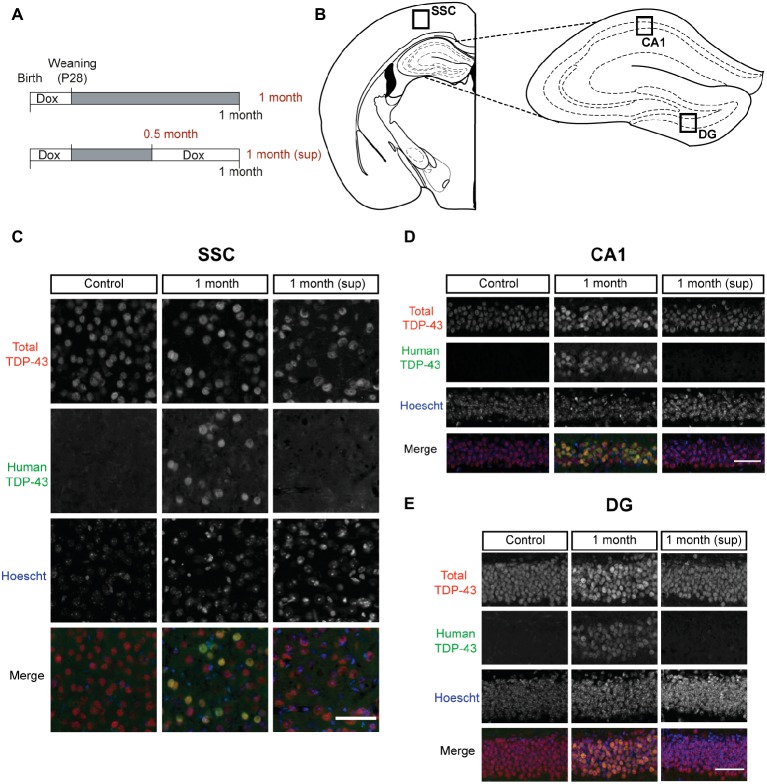
TDP-43 expression pattern in inducible TDP-43-WT transgenic mice. **(A)** Experimental design: transgene expression was inactive until postnatal day 28 by treatment with Dox. For the suppression protocol, mice were treated again with Dox at 0.5 month post-weaning to suppress transgene expression for 2 weeks. The behavioral analysis (motor, cognitive, and social) was performed at 0.5 month post-weaning (0.5 month mice) and at 1 month post-weaning [1 month (sup) mice]. The results on these mice were compared with mice in which transgene expression was preserved until 1 month post-weaning (1 month mice). **(B**–**E)** Expression of human TAR DNA-binding protein 43 (TDP-43) in tTA/WT12 mice. **(B)** Schematic diagrams (adapted from Paxinos and Franklin, 2008) showing different hTDP-43-expressing brain areas (indicated by the labeled boxes) such as somatosensory cortex (SSC), hippocampal cornus ammonis 1 (CA1), and dentate gyrus (DG). **(C–E)** Double immunofluorescence of total TDP-43 (human + mouse TDP-43) and human TDP-43 in representative coronal brain sections of control, tTA/WT12 1 month and tTA/WT12 1 month (sup) mice. High power micrographs of boxed areas in B are shown: SSC **(C)**, hippocampal CA1 region **(D),** and DG **(E)**. Scale bar: 50 μm.

Mice were treated with 0.2 mg/ml Dox (Doxycycline Hyclate, sc-204734A, Santa Cruz Biotechnology) in drinking water to avoid prenatal and postnatal developmental effects of transgene expression. hTDP-43 expression was induced by switching mice to regular drinking water (without Dox) at weaning (postnatal day 28). Mice were analyzed at different time points (Figure 1A).

Mice were screened for the presence of the transgene using genomic DNA isolated from ear biopsies. PCR amplification was done with the following primers: TDP-forward (TTGGTAATAGCAGAGGGGGTGGAG), MoPrP-reverse (TCCCCCAGCCTAGACCACGAGAAT), Camk2a-tTA-forward (CGCTGTGGGGCATTTTACTTTAG), and Camk2a-tTA-reverse (CATGTCCAGATCGAAATCGTC) as previously described (Igaz et al., 2011; Alfieri et al., 2014). To homogenize genetic background and minimize variability, the TDP-43-WT12 transgenic line used in these experiments was established by crossbreeding with C57BL/6J mice for >10 generations. For both non-transgenic and transgene-expressing groups, animals of either sex were included in all experimental groups.

### Transgene Suppression Protocol

For suppression experiments, mice were treated again with 0.2 mg/ml Dox in drinking water at 0.5 month after weaning to suppress transgene expression for 2 weeks, as indicated in Figure 1A. Animals were analyzed before the transgene suppression at 2 weeks after weaning (0.5 month mice) and after transgene suppression, at 1 month after weaning [1 month (sup) mice]. These mice were also compared with animals in which transgene expression was maintained until 1 month (1 month mice) after weaning.

### Behavioral Studies

All behavioral tasks were performed during the light phase (lights on at 7 a.m.; lights off at 7 p.m.) with the exception of the Y-maze spontaneous alternation, which was conducted during the initial dark phase (7:00 p.m to 9:00 p.m.) to maximize exploratory behavior and consistently obtain a high number of arm visits. Animals were allowed to habituate in the experimental room (with attenuated light and sound) for at least 1 h before the tests. All tests were recorded through a video camera mounted above the experimental room (unless noted) and mouse position was analyzed by automatic video tracking software (ANY-maze, Stoelting Co.). All mazes and objects used in behavioral analysis were cleaned with 10% ethanol between test sessions and sanitized with 70% ethanol at the end of the day.

In agreement with what we previously demonstrated in experiments with TDP-43-∆NLS and TDP-43-WT transgenic mice (Alfieri et al., 2014, 2016), all non-bigenic offspring (non-transgenic and both single transgenic mice) exhibited similar behavioral responses. Thus, for all subsequent behavioral tests and other experimental analyses, we grouped these genotypes under the control group to compare against bigenic (tTA/WT12) mice.

#### Y-Maze Spontaneous Alternation Test

The horizontal Y-shaped maze consisted of three identical arms of transparent Plexiglas (43 cm × 4 cm × 12.5 cm) placed at 120° angles to each other (Belforte et al., 2010; Alfieri et al., 2014). The test was performed in a room with visual clues and controlled illumination (30 lux), as previously described (Alfieri et al., 2014, 2016). Animals were placed at the end of one arm facing the center and allowed to freely explore the maze for 8 min without training, reward, or punishment. All activities were recorded with a computer-linked video camera mounted above the maze. Mouse position was detected by automatic video tracking (ANY-maze, Stoelting). An alternation was defined as consecutive entrances into each of the three arms without repetition. The percentage of spontaneous alternation was calculated as the number of alternations divided by the possible alternations [(# alternations)/(total arm entries − 2) × 100]. Total entries were scored as an index of ambulatory activity in the Y maze and mice with scores below 12 were excluded from this test. All mice were tested between 7:00 p.m. and 9:00 p.m. (dark phase) to maximize exploratory behavior (Belforte et al., 2010).

#### Social Interaction Test

The test apparatus comprised a black Plexiglas rectangular box (40.6 cm × 15 cm × 23 cm) divided into three interconnected chambers. The floor of the apparatus was covered with clean bedding. The task was performed as previously described (Alfieri et al., 2014, 2016). For the habituation phase, two identical cylinders of transparent Plexiglas (7 cm diameter, 14 cm tall) with multiple small holes (0.5 cm diameter) to allow olfactory, visual, and auditory interaction, were placed in each one of the end chambers. Then, the test mouse was placed in the central chamber for 5 min and allowed to explore the entire social interaction apparatus. During the test phase, a black object (non-social stimulus) was placed into one cylinder on the “non-social” chamber and a stimulus mouse (a 21–26 days old C57BL/6J male mouse) was placed into the cylinder on the “social” chamber. The test mouse was placed again in the central chamber and allowed to freely explore the apparatus for 10 min. Sniffing time for the social and non-social stimulus was manually scored. Mouse position and time spent in each chamber were analyzed by automatic video tracking (ANY-maze, Stoelting).

#### Open Field Test

To analyze general locomotion and exploratory behavior in a novel environment, we performed the open field test as previously described (Alfieri et al., 2014, 2016). The open field apparatus consisted of a transparent Plexiglas (40 cm × 40 cm × 40 cm) arena with a white floor virtually divided into two zones: periphery and center (comprising 50% of the total area centered). The test mouse was able to explore the novel environment for 20 min. Total distance and center distance traveled by the animal were analyzed. Time bin analysis (every 5 min) was also used. Room illumination was kept at 50 lux. Mouse position was determined by automatic video tracking (ANY-maze, Stoelting).

#### Accelerated Rotarod

For the assessment of motor coordination and balance, we used a rotarod apparatus (Ugo Basile, model 7600). The test was performed as previously described (Alfieri et al., 2016). Briefly, the accelerating rotarod test was set at 4–40 rpm over 300 s, and four trials per test were performed, with a 2-min interval between trials. The latency to fall off from the rotarod was automatically quantified. Mice that rotated passively were removed from the apparatus and scored as fallen.

#### Clasping Phenotype

The presence of clasping was evaluated as previously described (Igaz et al., 2011; Alfieri et al., 2014). hTDP-43-WT12 transgenic mice and age-matched control mice were suspended by the tail 30 cm over an open cage for 30 s. A positive clasping posture was noted for mice that clasped their limbs within 5 s of suspension while maintaining the clasping posture until lowered to the cage.

#### Hanging Wire Grip Test

Grip strength was studied using a standard wire cage and performed as previously described (Alfieri et al., 2014, 2016). Briefly, the mouse was placed on the top of the lid, then the lid was shaken lightly to cause the mouse to grip the wires and next turned upside down. The upside-down lid was held at a height of 20 cm, and the latency to fall off the wire lid was registered. A 60-s cutoff time was used.

### Brain Tissue Collection

All animals were deeply anesthetized with intraperitoneal administration of 5% chloral hydrate (1 ml/30 gr). Next, mice were perfused transcardially with ice-cold PBS (0.1 M, pH 7.4) supplemented with 10 U/ml heparin. The brains were immediately extracted and fixed overnight by immersion in 4% paraformaldehyde (PFA), and then cryoprotected in 10 and 30% sucrose in PBS for immunofluorescence analyses.

### Immunofluorescence

Fixed frozen hemispheres were cryosectioned (50 μm) on a sliding freezing microtome (SM 2010R; Leica). The brain slices were stored at −20°C in cryoprotecting solution (50% glycerol, 50% PBS). Double immunofluorescence was performed as follows: the coronal free-floating sections were washed 2 × 5 min with PBS, permeabilized with 1% Triton X-100 in PBS for 1 h, and blocked for 1 h with 0.3% Triton X-100 and 5% goat serum in PBS. The primary antibodies (diluted in 0.3% Triton X-100 and 3% goat serum in PBS) were incubated overnight at 4°C with the indicated dilutions: polyclonal rabbit anti-TDP-43 (as described previously in Igaz et al., 2008) 1:30,000 and monoclonal mouse anti-hTDP-43 (60019-2; Proteintech) 1:10,000. After washing 2 × 5 min with PBS, secondary antibodies conjugated with Alexa Fluor 488 (Invitrogen) and rhodamine (Jackson Laboratories) diluted in 0.3% Triton X-100 and 5% goat serum in PBS were incubated for 4 h at room temperature. Nuclear counterstaining was performed with Hoechst 33342 (2 μg/ml; Sigma). Sections were mounted using 30% glycerol in PBS on gelatin-coated slides. The images were obtained by a Zeiss Axio Imager 2 microscope equipped with APOTOME.2 structured illumination, using a Hamamatsu Orca Flash 4.0 camera.

### Statistical Analysis

Statistical tests were performed as follows, as described in the text and figure legends for each dataset. Student’s *t* test was used when comparing only two groups on one behavioral measure. Repeated measures (RM) two-way analysis of variance (ANOVA) followed by Newman-Keuls multiple comparison *post hoc* test, when comparing three or more groups in the social interaction experiments. RM-ANOVA followed by Bonferroni’s multiple comparisons *post hoc* test was used for accelerated rotarod. Fisher exact test was performed for clasping analysis. When non-parametric tests were required (hanging wire grip test), Mann-Whitney *U* test was used. Statistical analysis of behavioral tests was performed using PRISM 6 (Graph Pad software) or Statistica 7 (Stat Soft). Data are presented as mean values ± SEM. A *p* < 0.05 was considered statistically significant.

## Results

In a recent work, we performed a detailed characterization of the behavioral changes occurring in hTDP-43-WT mice (Alfieri et al., 2016). Using an induction protocol that avoids developmental and early postnatal deficits (Igaz et al., 2011), we showed that post-weaning expression of hTDP-43 in forebrain neurons for 1 month led to social and cognitive phenotypes in the absence of clear motor abnormalities. The main goals of this work were to understand whether these social and cognitive changes occur earlier following hTDP-43 expression and to define if the suppression of transgene expression might prevent or reverse the behavioral deficits.

tTA/WT12 bigenic mice or control littermates were induced at weaning (postnatal day 28) by removing Dox from their water supply (Figure 1A). Paralleling our studies using mice that express a cytoplasmic form of TDP-43 (hTDP-43-∆NLS) under the same promoter system (Alfieri et al., 2014), we defined a protocol that induces transgene expression for 2 weeks, and then, we suppressed hTDP-43 expression by treating mice for two additional weeks with Dox. We analyzed these animals at two time points, thus defining two experimental groups: mice after 2 weeks of Dox removal (0.5 month) and mice with subsequent transgene suppression due to treatment with Dox for additional 2 weeks, termed 1 month (sup) (Figure 1A). In this way, we can study both the installment/phenotype development at very early time points and the effect of transgene suppression on behavioral performance. This analysis, combined with our data from tTA/WT12 mice induced for 1 month (Figure 1A; Alfieri et al., 2016), allows us to study and interpret the susceptibility of different behavioral domains to transgene suppression.

In order to assess proper regulation of transgene expression within our experimental timeline, we performed double immunofluorescence studies (Figures 1C–E). We used a polyclonal antibody (referred as total TDP-43) that reacts to both endogenous (mouse) and transgenic (human) forms of TDP-43, and a monoclonal antibody that only recognizes the human isoform of the TDP-43 protein (in this case, human TDP-43-WT), termed human TDP-43 (hTDP-43). Representative micrographs from forebrain regions (see diagram in Figure 1B), including the somatosensory cortex (SSC, Figure 1C), hippocampal cornus ammonis 1 (CA1) region (Figure 1D), and Dentate Gyrus (DG, Figure 1E), show that bigenic tTA/WT12 mice correctly express transgenic human TDP-43 in the nucleus after 1 month of Dox removal (1 month group). On the contrary, bigenic mice from the 1 month (sup) group are almost completely devoided of hTDP-43 immunoreactivity. As expected, mice from the control group only show signal for the total TDP-43 antibody but no hTDP-43 staining (Figures 1C–E). Interestingly, total TDP-43 staining in the 1 month (sup) animals (but not in the control or 1 month groups) showed that a subset of cortical and hippocampal cells display altered nuclear morphology and/or nuclear TDP-43 distribution, suggesting that the mechanisms for coping with hTDP-43 overexpression and recovery might differ. These results demonstrate both robust nuclear expression of the transgene after Dox removal and proper suppression of hTDP-43 expression following re-installment of Dox treatment.

### Suppression of TDP-43-WT Expression Prevents Installment of Early Cognitive Deficits

**Figure 2:**
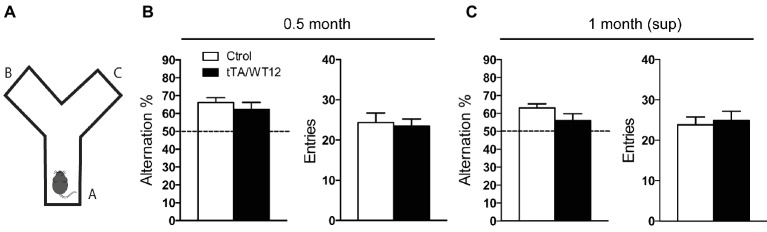
Short-term hTDP-43-WT suppression prevents installment of working/spatial memory deficits. **(A–C)** Y-maze spontaneous alternation task. **(A)** Scheme of the Y-maze. **(B,C)** Mice were allowed to explore the maze freely for 8 min without training, reward, or punishment. Alternation behavior (defined as a consecutive entrances into each of the three arms without repetition) and total arms entries, as an index of locomotion activity, were quantified. **(B)** 0.5 month mice Y-maze performance (n = 12, 11 for control and bigenic 0.5 month mice, respectively). **(C)** 1 month (sup) mice performance (n = 12, 10 for control and bigenic 1 month (sup) mice, respectively).

We have recently demonstrated that post-weaning overexpression of hTDP-43-WT during 1 month leads to cognitive deficits in our inducible tTA/WT12 mouse model (Alfieri et al., 2016). These phenotypes include alterations not only in object recognition memory but also in spatial working memory, as assessed by the object recognition test and the Y-maze spontaneous alternation test, respectively. These cognitive tasks rely on cortical (perirhinal, prefrontal) and hippocampal functional integrity (Lalonde, 2002; Warburton and Brown, 2010), and these areas widely express the transgene (Figure 1; Igaz et al., 2011). Since typical clinical features of FTD patients include alterations of prefrontal-dependent executive functions (Seelaar et al., 2011), we used the Y-maze task to monitor the impact of short-term hTDP-43 overexpression in this type of behavior (Figure 2A). The Y-maze test relies on the animal’s preference to explore a new arm of the maze rather than returning to a previously visited arm (Lalonde, 2002). When tTA/WT12 mice were tested after 0.5 month of induction, control mice showed spontaneous alternation percentages avoiding the previously visited arms and bigenic mice were indistinguishable from control mice, indicating normal working memory in both groups (*t*(21) = 0.8096, *p* = 0.4273; Figure 2B, left panel). Importantly, locomotion (estimated by the number of arm entries) was similar between groups (*t*(21) = 0.2947, *p* = 0.7711; Figure 2B, right panel). These data, together with our results showing that tTA/WT12 mice displayed altered performance in the Y-maze test 1 month post-induction (Alfieri et al., 2016), indicate that cognitive deficits (specifically, spatial working memory) begin to occur in the 0.5–1 month time window of transgene expression in this mouse model.

To evaluate if short-term (2 weeks) transgene suppression could prevent the installment of this deficit, we reintroduced Dox in the drinking water of these animals after the 0.5 month time point and assessed Y-maze performance 1 month post-weaning, in a group termed 1 month (sup) (see timeline in Figure 1A). Student’s *t* test analysis established that bigenic animals from the 1 month (sup) group did not show significant differences compared with control mice (*t*(20) = 1.658, *p* = 0.1128; Figure 2C, left panel). Again, the total number of arm entries was similar between groups (*t*(20) = 0.3583, *p* = 0.7238; Figure 2C, right panel). In summary, these data demonstrate that short-term suppression of transgene expression can prevent cognitive abnormalities in young TDP-43-WT mice.

### TDP-43-WT Mice Develop Sociability Abnormalities Very Rapidly After hTDP-43 Induction and They Persist After Transgene Suppression

**Figure 3:**
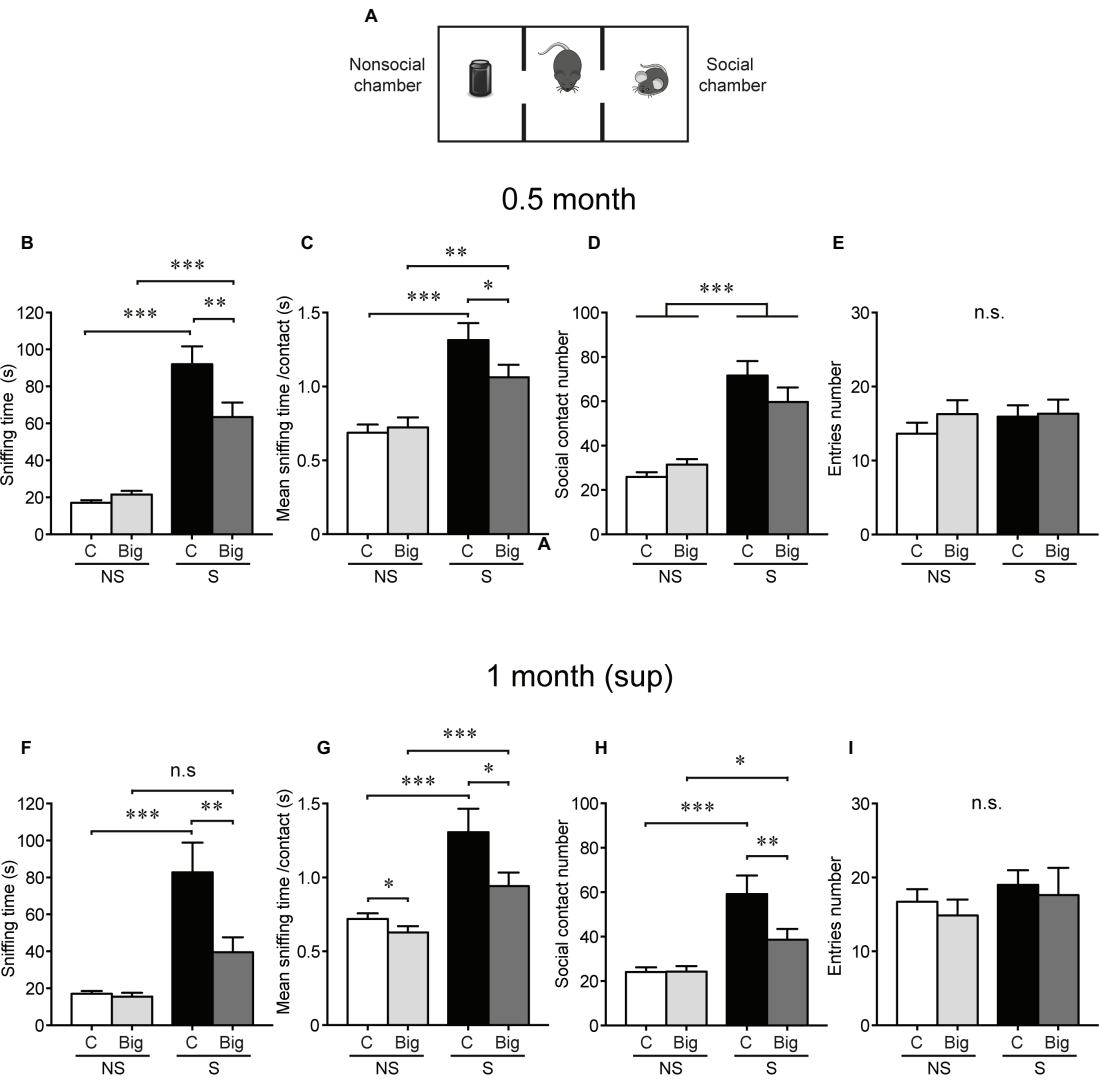
tTA/WT12 mice display early sociability deficits, which persist after transgene suppression. **(A)** Scheme of three-chamber social interaction apparatus, a rectangular box made of a black Plexiglass divided in three interconnected chambers. Social behavior was analyzed at 0.5 months off Dox (0.5 month mice) **(B–E)**, and after 2 weeks of Dox transgene suppression treatment [1 month (sup)] **(F–I)**. **(B,F)** The time spent sniffing the social (S; P21–P28 male mouse) or the non-social (NS; black plastic object) stimulus during a 10 min session (test phase) was recorded. **(C,G)** Mean sniffing time per contact. **(D,H)** Number of social contacts. **(E,I)** Total entries in each chamber. *p < 0.05, **p < 0.01, ***p < 0.001, two-way RM-ANOVA/Newman-Keuls *post hoc* test, except for **(G)**, significance of main effects. *n* = 11, 15 for control and bigenic 0.5 month mice, respectively; *n* = 10, 13 for control and bigenic 1 month (sup) mice, respectively. n.s., not significant. Data represent mean ± SEM.

Within the spectrum of clinical presentations of TDP-43 proteinopathies, deficits in social behavior are a conspicuous feature of FTD patients (Desmarais et al., 2017). Moreover, we and others have shown that altered sociability is a feature of different animal models of FTD, including those based on manipulations of TDP-43, tau, fused in sarcoma (FUS), progranulin, and CHMP2B (Ghoshal et al., 2012; Filiano et al., 2013; Alfieri et al., 2014, 2016; Koss et al., 2016; Vernay et al., 2016; Shiihashi et al., 2017). Specifically, we demonstrated that both inducible transgenic mice expressing either cytoplasmic (TDP-43-∆NLS) or nuclear (TDP-43-WT) form of human TDP-43 show altered early social phenotypes, 1 month post-induction (Alfieri et al., 2014, 2016). At this time point, bigenic tTA/WT12 mice display decreased performance in the three-chamber social interaction test (Figure 3A) respective to control animals (Alfieri et al., 2016), although to a lesser degree than TDP-43-∆NLS mice (Alfieri et al., 2014).

To determine whether this phenotype was detectable right before starting the suppression protocol, we studied sociability in tTA/WT12 mice 0.5 month after hTDP-43 induction. As expected, control mice spent more time interacting with the demonstrator mouse (S, social stimulus) than with the inanimate object (NS, non-social stimulus). Bigenic mice showed a deficit in social interaction evidenced by a significant decrease in time spent in direct social exploration of a conspecific demonstrator (sniffing time, RM-ANOVA, interaction *F*_(1,24)_ = 6.32, *p* = 0.019; Figure 3B). This reduced social activity results from a significant reduction in the mean sniffing time per contact (RM-ANOVA, interaction *F*_(1,24)_ = 4.60, *p* = 0.042; Figure 3C) with no significant differences in the number of social contacts among social conditions (RM-ANOVA, S-NS preference *F*_(1,24)_ = 46.98, *p* < 0.0001; Figure 3D). Decreased social interaction cannot be attributed to an altered motor function (non-significant differences in the total number of chamber entrances, RM-ANOVA, S-NS preference *F*_(1,24)_ = 0.57, *p* = 0.46, genotype *F*_(1,24)_ = 0.54, *p* = 0.47, interaction *F*_(1,24)_ = 0.51, *p* = 0.48, Figure 3E, and total traveled distance, 16.95 ± 2.57 vs. 14.80 ± 1.77 m for control and bigenic mice, respectively; *t*(24) = 0.7137, *p* = 0.4823, Student’s *t* test) or to general deficits in novelty exploration (both groups interact similarly with the novel object, compare non-social control vs. non-social bigenic groups in Figures 3B–D).

Next, we assessed the effect of turning off hTDP-43 expression on social behavior. Notably, transgene suppression in the 1 month (sup) group did not cause any improvement in social interaction in bigenic mice respective to controls (RM-ANOVA, interaction *F*_(1,21)_ = 6.41, *p* = 0.019; Figure 3F). Moreover, the social phenotype showed a tendency to worsen after transgene suppression, since both the mean contact duration (RM-ANOVA, S-NS preference *F*_(1,21)_ = 27.76, *p* < 0.0001; genotype *F*_(1,21)_ = 5.60, *p* = 0.027; Figure 3G) and the total contact number (RM-ANOVA, interaction *F*_(1,21)_ = 4.75, *p* = 0.041; Figure 3H) now showed significantly lower values in bigenic mice respective to controls. The number of entrances was equivalent in both groups in both social and non-social sides (RM-ANOVA, S-NS preference *F*_(1,21)_ = 2.76, *p* = 0.11; genotype *F*_(1,21)_ = 0.21, *p* = 0.64; interaction *F*_(1,21)_ = 0.02, *p* = 0.88; Figure 3I), as was the interaction with the novel object (non-social control vs. bigenic comparison in Figures 3F,H) and the total traveled distance (16.57 ± 1.64 vs. 11.75 ± 1.74 m for control and bigenic mice, respectively; *t*(21) = 1.967, *p* = 0.063, Student’s *t* test). These results show that TDP-43-WT mice very rapidly develop alterations in social behavior, and these cannot be ameliorated by short-term suppression of transgene expression. Moreover, they support the idea that diverse behavioral domains present differential susceptibility to hTDP-43 overexpression and subsequent renormalization of TDP-43 levels.

### Preserved Motor Behavior in TDP-43-WT Mice After Short-Term Transgene Overexpression and Upon Dox Treatment

Although TDP-43 proteinopathies include the FTD/ALS spectrum of disorders, only a subset of FTD cases present with motor abnormalities. In case of tTA/WT12 mice, we described a progressive motor phenotype that slowly develops with increased time of hTDP-43 expression (Alfieri et al., 2016). In this mouse model, motor deficits are virtually absent at 1 month post-induction but gradually emerge and are clearly present after 12 months of transgene expression.

**Figure 4:**
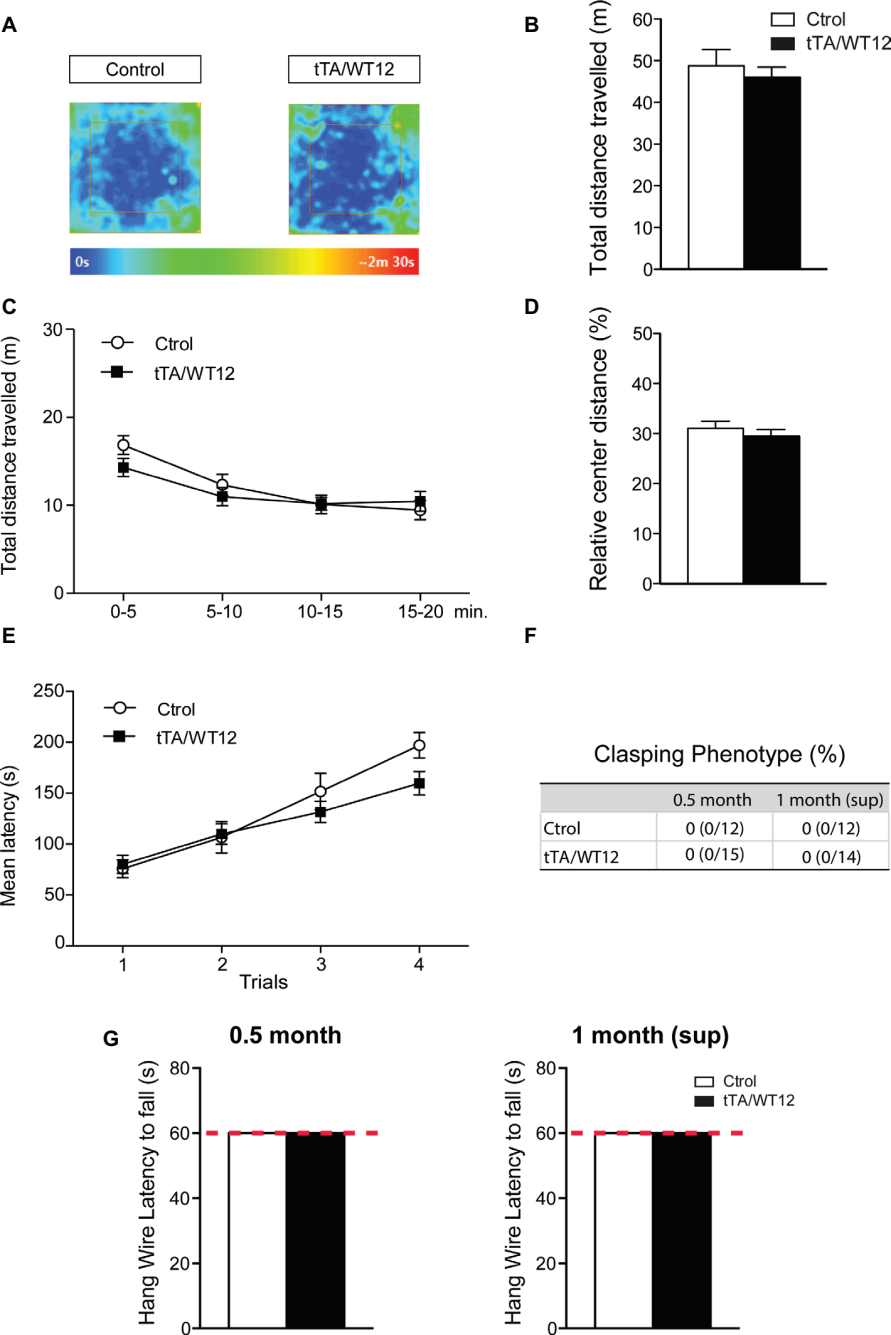
Motor domain remains unaltered after 2 weeks of suppression of hTDP-43-WT transgene. **(A–D)** Open-field task; locomotor and exploratory behaviors were preserved in suppressed hTDP-43-WT mice. **(A)** Occupancy plot. Representative heat maps for time spent by subject mice during the entire session are shown. The heat map was constructed based on body position. **(B)** Total distance traveled. **(C)** Total distance traveled in time segments of 5 min. **(D)** Relative center distance. No significant differences were found between controls and bigenic animals in locomotion or exploration. **(E)** Motor coordination and balance were not affected after suppression of hTDP-43-WT expression. Accelerated rotarod performance (4–40 rpm/5 min): four trials per test were performed during the test day with a 2-min interval between trials. Latency to fall off the apparatus was recorded. **(F–G)** No signs of spasticity or motor strength deficits are detected in suppressed hTDP-43-WT mice. **(F)** The absence of clasping phenotype in both 0.5 month and 1 month (sup) mice. Percentage of animals positive for abnormal clasping and number of animals positive/total tested are shown. **(G)** Hanging wire grip test. Grip strength was assessed using a standard wire cage turned upside down. The latency to fall off the wire lid was quantified; a 60-s cutoff time was used. No significant differences were found between control and bigenic animals [*p* > 0.05 Student’s *t* test in **(B,D)**, Mann-Whitney *U* test in **(G)**, repeated-measures ANOVA in **(C,E)**, Fisher’s exact test in **(F)**]. *n* = 12, 15 for control and bigenic 0.5 month mice, respectively; *n* = 12, 14 for control and bigenic 1 month (sup) mice, respectively. Data represent mean ± SEM.

We assessed general motor function and exploratory activity in the 1 month (sup) group using the open field test (Figures 4A–D). In agreement with the data from 1 month induced mice (Alfieri et al., 2016), bigenic tTA/WT12 mice that underwent the suppression protocol traveled the same distance as control animals (*t*(24) = 0.6150, *p* = 0.5444; Figures 4A,B). A more detailed analysis of this parameter, dividing the session in 5 min time bins, indicated that there was no averaging effect and both groups performed similarly in each of the time segments (Figure 4C). Moreover, relative center distance in this task was also indistinguishable from controls (*t*(24) = 0.7907, *p* = 0.4369; Figure 4D). Other parameters showed no differences (Student’s *t* test) between bigenic and control groups, including average speed (4.07 ± 0.33 vs. 3.83 ± 0.21 cm/s for control and TDP-43-WT12 mice, respectively; *t*(24) = 0.6294, *p* = 0.5351), maximum speed (60.2 ± 8.9 vs. 54.7 ± 4.2 cm/s for control and TDP-43-WT12 mice, respectively; *t*(24) = 0.5888, *p* = 0.5615), and percentage time in center (18.83 ± 1.67 vs. 16.81 ± 1.68% for control and TDP-43-WT12 mice, respectively; *t*(24) = 0.8465, *p* = 0.4057).

Next, we evaluated motor coordination and balance in TDP-43-WT animals using the accelerated rotarod test. Both bigenic and control mice behaved similarly in the 1 month (sup) paradigm (repeated-measures ANOVA, *F*_(1,24)_ = 0.9200, *p* = 0.3470 for group; *F*_(3,72)_ = 44.09, *p* < 0.0001 for trial; *F*_(3,72)_ = 2.342, *p* = 0.0804 for interaction; Figure 4E). We also performed an analysis of limb clasping reflex phenotype. Both control and bigenic animals extended their limbs normally when being suspended by their tails, after 0.5 month of induction and subsequently after transgene suppression (Figure 4F). In addition, TDP-43-WT mice displayed indistinguishable latencies to fall in a hang wire test in the 0.5 month induction and 1 month (sup) groups, indicating intact grip strength (Figure 4G).

These data indicate that: (1) motor abnormalities are not present after 0.5 month of transgene expression, consistent with our results after 1 month of hTDP-43 overexpression (Alfieri et al., 2016) and (2) the suppression protocol had no unspecific effects on motor behavior, since locomotor/exploratory behavior, motor coordination, limb clasping, and grip strength were indistinguishable from controls.

**Figure 5:**
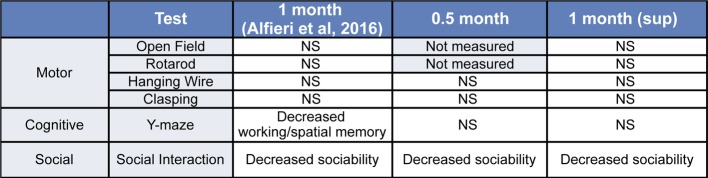
Summary of the behavioral phenotypes observed in tTA/WT12 mice after 0.5 or 1 month (from Alfieri et al., 2016) of hTDP-43 expression and in the 1 month (sup) group. NS: non-significant differences respective to control mice.

In summary, we present evidence that defines the early time course of behavioral impairments and establish the susceptibility of different behavioral domains to transgene suppression in tTA/WT12 mice (Figure 5).

## Discussion

Animal models are invaluable tools to understand the pathological basis of neurodegenerative diseases such as FTD and ALS. In this study, we explored the behavioral consequences of suppressing hTDP-43 expression in our inducible tTA/WT12 mice.

The identification of disease-related molecules, the discovery of pathogenic pathways, and the targeting of pathogenic proteins are all critical steps to establish therapeutic strategies for incurable diseases, including neurodegenerative conditions. A major breakthrough was the discovery of TDP-43 as a pathological hallmark of ALS and FTD (Arai et al., 2006; Neumann et al., 2006). Mutations in more than 25 genes have been shown to cause ALS and FTD, and it is noteworthy that most of these mutant proteins participate in two intracellular machineries: the RNA and protein quality control systems (Ito et al., 2017).

TDP-43 belongs to a large family of RNA-binding proteins (RBPs) that have been shown to have multiple links with disease pathogenesis. Of specific relevance for neurodegenerative diseases, a prominent theory in the field states that neurons are particularly vulnerable to disruption of RBP dosage and dynamics (Conlon and Manley, 2017; Cookson, 2017). In particular, recent progress investigating the genetics of the FTD/ALS disease spectrum has shown that at least seven RBPs have been identified with disease-related mutations. These include TDP-43, FUS, the heterogeneous nuclear ribonucleoproteins (hnRNPs) hnRNPA1 and hnRNPA2B1, T cell intracytoplasmic antigen (TIA1), TATA box-binding protein-associated factor 15 (TAF15), and Ewing sarcoma breakpoint region 1 (EWSR1) (reviewed in Ito et al., 2017). This avalanche of information resulted in the development and characterization of multiple animal models of FTD/ALS based on the altered expression of these proteins (Lutz, 2018). These include TDP-43 models in both invertebrate (*C. elegans*, *Drosophila*) and vertebrate (mouse, rat) organisms (reviewed in Picher-Martel et al., 2016). In addition to traditional transgenic and knockout technology, approaches such as application of CRISPR/Cas9 and viral transgenesis provide exciting alternative avenues to explore.

Inducible animal models based on the tet-tTA system have been used to assess the neuropathological and behavioral impact of expression of different neurodegeneration-associated proteins, including TDP-43, tau, APP, α-synuclein, SCA1, SCA3, and huntingtin (Yamamoto et al., 2000; Zu et al., 2004; Jankowsky et al., 2005; Nuber et al., 2008; Boy et al., 2009; Sydow et al., 2011; Walker et al., 2015). However, only a few studies provided evidence for selective behavioral vulnerability to transgene suppression in neurodegenerative disease models.

Transgenic mice expressing TDP-43 carrying a pathogenic A315T mutation in CNS neurons display early motor and anxiety-like phenotypes that are reversible on Dox treatment, while memory impairments persist after transgene suppression (Ke et al., 2015). Our previous work in a mouse model expressing a cytoplasmic form of TDP-43 (TDP-43-∆NLS) demonstrated a differential response to transgene suppression, reversing the early motor and cognitive defects but having no effect of the sociability abnormalities developed by these mice (Alfieri et al., 2014). In light of these recent examples, the evaluation of differential behavioral susceptibility to TDP-43-related dysregulation arises as a relevant parameter for understanding the progression of neurodegenerative disease manifestations, particularly in TDP-43 proteinopathies.

Our results show that cognitive performance, as evaluated in the Y-maze test, remains unaltered after 2 weeks of wild-type hTDP-43 expression (0.5 month group). By contrast, we previously reported that expression of TDP-43-∆NLS using the same promoter system and induction protocol leads to a clearcut decrease in Y-maze alternation, reaching levels indistinguishable from chance and thus indicating severe deficits in spatial working memory (Alfieri et al., 2014). While those animals recover their cognitive capacity when suppressed in the 1 month (sup) group, we interpret the normal levels of tTA/WT12 mice Y-maze performance in 1 month (sup) mice as evidence that we are preventing the development of the working memory phenotype described in hTDP-43-WT mice with continuous expression for 1 month (Alfieri et al., 2016). However, we want to stress here that we are not comparing the degree of cognitive dysfunction between the different expression time points (0.5 and 1 months) and the 1 month (sup) groups, but qualitatively assessing if a phenotype is present. The different onset time for cognitive deficits in our two inducible models highlights the fact that TDP-43-WT mice have a milder behavioral phenotype than TDP-43-∆NLS. This is also substantiated by the analysis of social and motor behavior.

In terms of social phenotype, our conditional hTDP-43-WT animal model recapitulates deficits that constitute a core feature of the clinical FTD/ALS spectrum, particularly in several subtypes of FTD (Shinagawa et al., 2006; Sado et al., 2009; Christidi et al., 2018). Decreased sociability is a recurrent feature of FTD patients, and the three-chamber social interaction test used here demonstrates that TDP-43-WT mice develop social deficits very rapidly, after only 2 weeks of transgene expression (0.5 month group). Contrary to what happened with cognitive performance, sociability cannot be rescued or preserved in these mice after short-term transgene suppression. Interestingly, TDP-43-∆NLS showed the same dynamics of social deficits, although the abnormalities were more profound in those mice (Alfieri et al., 2014). Altogether, these data indicate that the neuronal circuits underlying these two different behavioral domains are differentially affected in TDP-43-WT mice, which, when considered in the context of a similar result from TDP-43-∆NLS animals, suggest an exquisite vulnerability for TDP-43-elicited changes in social function.

Although motor abnormalities develop after at least 6 months of transgene expression in TDP-43-WT mice (Alfieri et al., 2016), we sought to establish that (1) motor deficits at 0.5 month of expression cannot explain the phenotypes in other behavioral domains evaluated (in addition to the internal controls within each test) and (2) re-introduction of Dox treatment did not alter motor performance, eliminating thus the possibility that the treatment necessary for transgene suppression could be interfering in the proper assessment or interpretation of behavioral tests performed in the 1 month (sup) group. This is an important point, since it has been reported that Dox treatment may have non-specific effects on certain behaviors, although the strain more resistant to these effects was C57BL/6J (Han et al., 2012) and our TDP-43 inducible mouse models are backcrossed to C57BL/6J for >10 generations to homogenize genetic background.

A limitation (but also an advantage) of this model is that, due to the pattern of expression provided by the driver transgenic line (CamKII promoter), which results in forebrain-enriched neuronal hTDP-43-WT expression, part of the phenotype can be restricted due to sparing of other regions (including spinal cord). However, there are other rodent models available with pan-neuronal TDP-43 expression (summarized in Picher-Martel et al., 2016) and comparison of our results with those can provide important clues on regional involvement.

Our results showing differential behavioral susceptibility to transgene suppression in our inducible TDP-43-WT mice stimulate further questions regarding the underlying mechanisms behind these differences. Additional research is warranted, exploring selective regional neurodegeneration and neuroinflammation, as well as the potential role of non-neuronal cells (i.e., astrocytes and microglia) as contributors to this phenotype.

In summary, we show here that not only TDP-43-WT mice recapitulate several core behavioral features of FTD/ALS spectrum of human pathology, but also these behavioral domains display a different time course of onset and sensitivity to transgene suppression. This information is particularly relevant to understand both the time windows of efficacy for potential treatments and the selectivity/sensitivity to TDP-43 dysregulation of the neural circuits underlying the clinical phenotypes displayed by FTD/ALS patients. We also consider that our inducible model is especially relevant to explore the etiology of TDP-43-related FTD, due to the predominant social/cognitive phenotype with early sparing of motor symptoms (which only appear after long-term expression of the transgene; Alfieri et al., 2016). The information provided from this and future studies using this animal model might shed light into the pathological mechanisms of TDP-43 proteinopathies and help devise potential therapeutic avenues for these devastating conditions.

## Ethics Statement

This study only involved animal subjects and no human subjects. The information regarding animal subject involvement is included in the manuscript (in the Materials and Methods section) as required.

## Author Contributions

PS and LI planned the design of the experiments and wrote the article. PS and GN carried out the experiments. PS and LI analyzed the data. All authors edited the manuscript. LI conceived and supervised all aspects of the project.

### Conflict of Interest Statement

The authors declare that the research was conducted in the absence of any commercial or financial relationships that could be construed as a potential conflict of interest.
